# Postoperative outcomes in robotic gastric resection compared with laparoscopic gastric resection in gastric cancer: A meta‐analysis and systemic review

**DOI:** 10.1002/hsr2.746

**Published:** 2022-08-16

**Authors:** Muhammad Ali, Yang Wang, Jianyue Ding, Daorong Wang

**Affiliations:** ^1^ Department of Gastrointestinal Surgery Northern Jiangsu People's Hospital Yangzhou China; ^2^ General Surgery Institute of Yangzhou Yangzhou University Yangzhou China; ^3^ Medical College of Yangzhou University Yangzhou China

**Keywords:** gastric cancer, laparoscopic gastrectomy, robotic gastrectomy

## Abstract

**Background:**

Robotic gastrectomy is a commonly used procedure for early gastric cancer and it also overcomes the limitation of laparoscopic. However, the complications of robotic gastrectomy (RG) still need to be assessed. Our study was designed to compare postoperative complications of RG with laparoscopic gastrectomy (LG).

**Materials and Methods:**

A meta‐analysis and systemic review were prospectively collected using the PubMed, Cochrane Library, and MEDLINE database of published studies by comparing the RG and LG with gastric cancer up to December 2021. To evaluate the postoperative outcomes, odds ratios were calculated for Dichotomous data and the mean difference with 95% confidence interval (CI) was calculated for continuous data, and measured by the random‐effect model.

**Results:**

Thirty‐two retrospective studies describing 13,585 patients (4484 RG and 9101 LG) satisfied the inclusion criteria. A statistically significant result was in blood loss (MD = −17.97, 95% Cl: −25.61 to 10.32, *p* < 0.001), Clavien−Dindo grade Ⅲ (odds ratio (OR) = 0.60, 95% CI: 0.48−0.76, *p* < 0.01), and harvested lymph node (MD = 2.62, 95% CI: 2.14−3.11, *p* < 0.001). There was no significant difference between robotic gastrectomy surgery (RGS) and laparoscopic gastrectomy surgery (LGS) regarding distal resection margin (DRM), proximal resection margin (PRM), conversion rate, anastomotic leakage, and overall complications.

**Conclusion:**

Having significant outcomes in Clavien–Dindo grade III, and blood loss, harvested lymph nodes are more common in RGS, and they also help in increasing the quality of life.

## 1 INTRODUCTION

Gastric cancer at present is still a leading cause of health problems and death due to cancer and it is the 5th most regularly identified cancer around the globe.^1^ The standard treatment for gastric cancer is surgical resection and open gastrectomy with lymph node dissection takes the main course in cancer treatment. Laparoscopic gastrectomy (LG) slowly spread worldwide and it was primarily informed in 1994 by Kitano et al.^2^ The comparison between open and laparoscopic surgery for gastric cancer of various clinical trials has shown similar outcomes.^3^, ^4^, ^5^ However, laparoscopic surgery shows some sort of limitations such as the reduced sense of touch, lack of flexibility, two‐dimensional motion, and narrow movement range of the instrument. Also, LG requires a long learning pathway in lymph node dissection and causes physical stress.^6^

In the meantime, Hashizume et al. were the first to perform robotic gastrectomy (RG) in 2003.^7^ Recently, RG has got an attractive technique to cure gastric carcinoma. A study of nonrandomized trials and meta‐analysis has definite that robotic gastrectomy surgery (RGS) over laparoscopic gastrectomy surgery (LGS) for gastric carcinoma can recover short‐term and long‐term results and assuming, it will improve the operative and surgical results.^8^ Distinguish studies between RG and LG have been informed of the patient's quality of life after minimal invasive surgery (MIS).^9^, ^10^, ^11^, ^12^, ^13^ These studies were not randomized controlled trials, so there is still controversy between RG and LG.

RGS has been stated to overcome the limitation of LGS and offers new features like wide‐ranging tremor filtering, HD vision magnification with 3D stereoscopic, self‐determination of device motion, upgraded surgeon dexterity, and a shorter learning curve.^13^, ^14^ Robotic gastrectomy was testified to be correlated with a lesser extent of operative blood loss and shorter clinic stay than LG.^15^, ^16^

Therefore, the postoperative complication of RGS comparison to LGS management in early‐stage gastric carcinoma had not been evaluated yet.

## 2 MATERIALS AND METHODS

### 2.1 Study strategy

**Figure 1:**
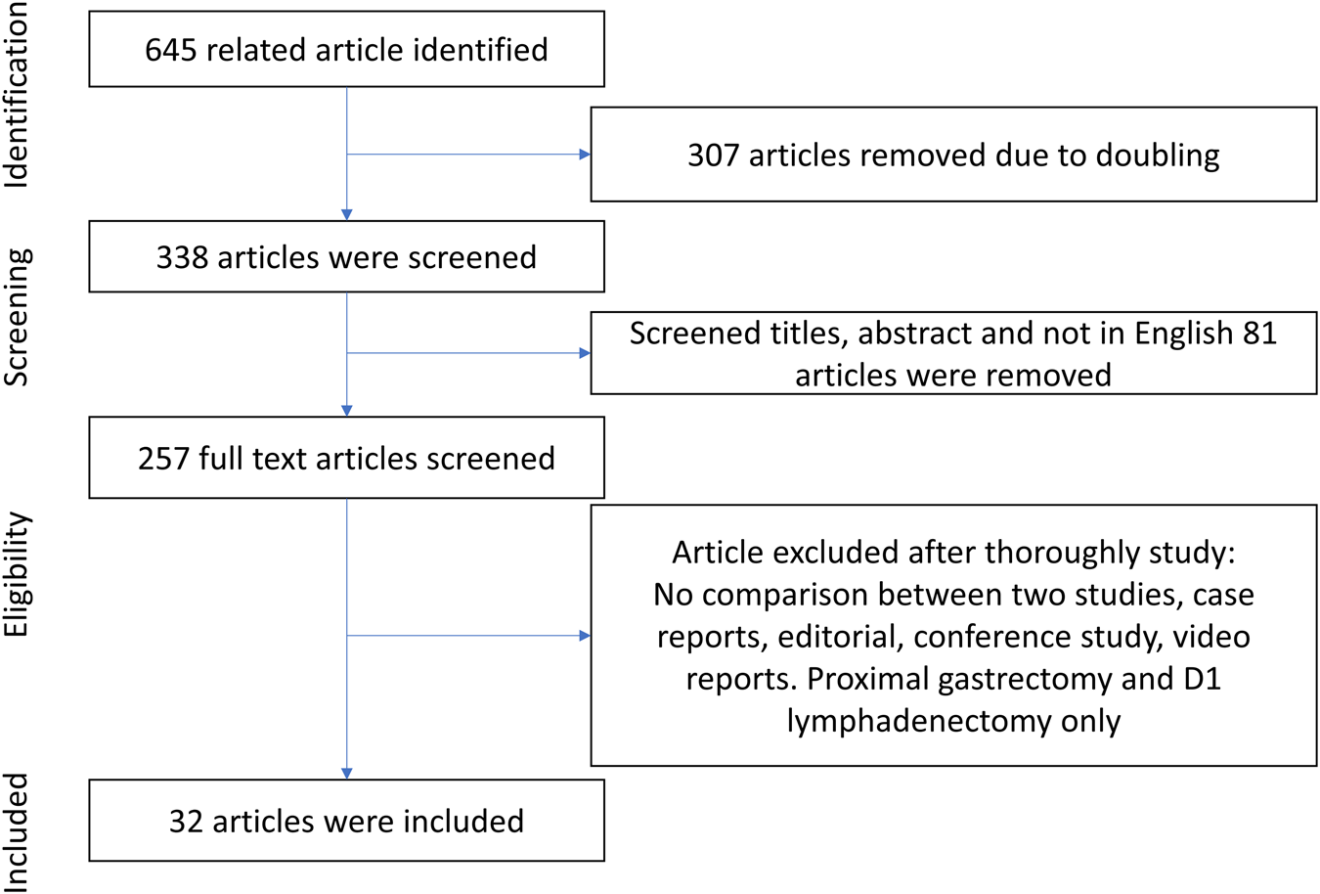
PRISMA diagram of the data collection method

We performed this study according to PRISMA and AMSTAR guidelines as shown in Figure 1. The MINORS measure indicates the value of detailed studies that are meticulously satisfactory to little heterogeneity concerning their quality, with an average score of 22 (range: 19–23) as present in Table 1.

**Table 1:** Studies elaboration in the meta‐analysis

n.	Author	Yr.	Region	Study period	Study design	Surgical extension	Sample size		MINOR	Ref.
							RG	LG		
1	Kim HI	2016	Korea	2011–2012	P	D, T	185	185	23	^9^
2	Suda	2015	Japan	2009–2012	R	D, T	88	438	22	^10^
3	Kim YW	2015	Korea	2009–2001	P	D	87	288	20	^11^
4	Kim KM	2012	Korea	2005–2010	P	D, T	436	861	23	^12^
5	Kang	2012	Korea	2008–2011	P	D, T	100	282	22	^13^
6	Eom	2011	Korea	2009–2010	P	D	30	62	22	^17^
7	Woo	2011	Japan	2005–2009	P	D, T	236	591	23	^18^
8	Yoon	2011	Korea	2009–2011	R	T	36	65	23	^19^
9	Son SY	2012	Korea	2007–2011	R	D, P, T	21	42	19	^20^
10	Hyun	2013	Korea	2009–2010	P	D, T	38	83	22	^21^
11	Kim HI	2013	Korea	2003–2009	P	D, T	172	481	22	^22^
12	Huang	2014	Taiwan	2008–2014	P	D, T	72	73	22	^23^
13	Junfeng	2014	China	2010–2013	R	D, P, T	120	394	23	^24^
14	Son T	2014	Korea	2003–2010	P	T	51	58	22	^25^
15	Han	2015	Korea	2008–2013	R	PPG	68	68	23	^26^
16	Lee	2015	Korea	2003–2010	P	D	133	267	21	^27^
17	Park	2015	Korea	2009–2011	P	D, T	145	612	19	^28^
18	Cianchi	2016	Italy	2008–2015	P	D	30	41	21	^29^
19	Hong	2016	Korea	2008–2015	P	D	232	232	22	^30^
20	Nakauchi M	2016	Japan	2009–2012	R	D, T	84	437	23	^31^
21	Okumura	2016	Japan	2003–2010	P	D, T	370	132	22	^32^
22	Shen	2016	China	2011–2014	R	D, T	93	330	21	^33^
23	Obama k	2017	Korea	2005–2009	P	D, T	315	525	23	^34^
24	Parisi	2017	Italy	2015–2016	P	D, T	151	151	21	^35^
25	Yang	2017	Korea	2009–2015	P	D, T	173	511	21	^36^
26	Gao Y	2018	China	2011–2014	P	D, P, T	163	339	21	^37^
27	Li Z	2018	China	2013–2017	P	D, T	112	112	23	^38^
28	Liu	2018	China	2017–2017	R	D, T	100	135	21	^39^
29	Lu	2018	China	2016–2017	P	D, T	101	303	20	^40^
30	Wang WJ	2018	China	2016–2018	P	D, T	223	223	23	^41^
31	Alhoassaini	2019	Korea	2005–2017	R	T	25	30	23	^42^
32	Kong	2019	China	2016–2017	R	D, P, T	294	750	23	^43^

Abbreviations: D, distal gastrectomy; P, prospectively collected data; T, total gastrectomy; Yr, year.

### 2.2 PICO

#### 2.2.1 Population

SCOPUS, Cochrane Library, and PubMed database for articles available until December 2021.

#### 2.2.2 Intervention

Having significant results in RGS, the Clavien–Dindo classification shows the most practicable and high‐quality approach for gastric cancer, with better surgical results due to the lesser number of patients in Clavien–Dindo grade Ⅲ.

#### 2.2.3 Comparison

We considered studies that compared RGS with LGS for gastric cancer and focused on postoperative complications.

#### 2.2.4 Outcome

Having significant outcomes in Clavien–Dindo grade III, and blood loss, harvested lymph nodes are more common in RGS, and they also help in increasing the quality of life.

#### 2.2.5 Inclusion criteria

Retrospective studies involving the RGS comparison with LGS for gastric carcinoma. English language full‐text article containing at least one of the following postoperative complications; blood loss, conversion rate, DRM, PRM, Clavien–Dindo grade Ⅲ, HLN, anastomosis leakage, and overall complication.

#### 2.2.6 Exclusion criteria

Articles about robotic or laparoscopic surgery that did not provide a comparison, evaluations that did not address complications, reviews, case reports, animal studies, and letters were all omitted.

### 2.3 Data collection and methodology

We systematically explored the literature by SCOPUS, Cochrane Library, and PubMed database for articles available until December 2021. Our research work included the keywords “Robotic gastrectomy,” “laparoscopic gastrectomy,” and “gastric cancer.” Our search is limited to humans and English language articles.

### 2.4 Statistical analysis

RevMan 5.4 was implemented for statistical meta‐analysis. Summative figures are arranged according to descriptive analysis and we set the confidence interval (Cl) at 95%. Outcomes are reported for dichotomous as odds ratios (OR) and 95% Cl through Mantel–Haenszel way and continuous variables as mean difference (MD) through generic inverse variance way. Continuous data, standard deviation (SD), and mean were reported in median and range. We set statistically significant at (*p* < 0.05), *Q* statistics were used to assess the treatment effects of heterogeneities, and *I* ^2^ was assessed for the total variation studies.

## 3 RESULTS

### 3.1 Studies and patient characteristics

A total of 645 articles were found from PubMed, Scopus, MEDLINE, and Cochrane Library with the search word “robotic gastrectomy,” “laparoscopic gastrectomy,” and “gastric cancer.” After screening articles, 307 were excluded because of doubling, screened titles, abstracts, and not in English 81 were removed, and a total of 257 full‐text articles were retrieved from which 257 articles with “no comparison between RG versus LG,” “proximal gastrectomy only,” “case reports,” “conference study,” “literature,” and “editorial” were removed. A flow illustration of the research course is shown in Figure 1. Thirty‐two retrospective studies were included, in which 13,585 patient descriptions are shown in Table 1 and postoperative complications are shown in Table 2. All the articles were nonrandomized trials, in which 4484 patients experienced RG for GC, while 9101 went through LG for GC.

**Table 2:** Postoperative complications

Postoperative outcome	Types of surgery	Observation	n.	Studies involved
Blood loss	RG	3921	103.6	27
	LG	8539	120.5	
Conversion rate	RG	2899	0.857	21
	LG	6415	2.62	
Overall complication	RG	4484	16.5	32
	LG	9101	34	
Anastomotic leakage	RG	3275	2.375	24
	LG	6890	5.83	
Clavien–Dindo Grade ≥ Ⅲ	RG	2851	5.9	19
	LG	5022	16.84	
DRM	RG	1468	6.69	11
	LG	3257	6.51	
PRM	RG	1519	4.51	12
	LG	3315	4.35	
HLN	RG	3813	39.77	28
	LG	7691	34.37	

Abbreviations: DRM, distal resection margin; HLN, harvested lymph node; LG, laparoscopic gastrectomy; n, mean; PRM, proximal resection margin; RG, robotic gastrectomy.

### 3.2 Postoperative outcomes

We set the statistical (*p* < 0.05), *Q* statistics were used to assess the treatment effects of heterogeneity, and *I* ^2^ was assessed for the total variation studies as shown in Table 3.

**Table 3:** Result of the meta‐analysis

Outcome	No. of studies	Sample size		Heterogeneity		Overall effect size	95% Cl of overall effect	*p* value
		LG	RG	*I* ^2^ (%)	*p* value			
Overall complications	32	9101	4484	33	0.04	OR = 0.87	0.77,0.98	0.02
Blood loss	27	8539	3921	89	<0.001	MD = −17.97	−25.61, −10.32	<0.001
Anastomosis leakage	24	6890	3275	0	0.98	OR = 0.86	0.63,1.18	0.35
Clavien–Dindo grade Ⅲ	19	5022	2851	29	0.12	OR = 0.60	0.48,0.76	<0.001
DRM	11	3257	1468	80	<0.001	MD = 0.13	−0.05,0.32	0.15
PRM	12	3315	1519	0	0.55	MD = 0.07	−0.07,0.22	0.30
HLN	28	7691	3813	77	<0.001	MD = 2.62	2.14,3.11	<0.001
Conversion rate	21	6415	2899	12	0.33	OR = 0.71	0.38,1.33	0.29

Abbreviations: Cl, confidence interval; DRM, distal resection margin; HLN, harvested lymph node; LG, laparoscopy gastrectomy; MD, mean difference; OR, odds ratio; PRM, proximal resection margin; RG, robotic gastrectomy.

### 3.3 Blood loss

**Figure 2:**
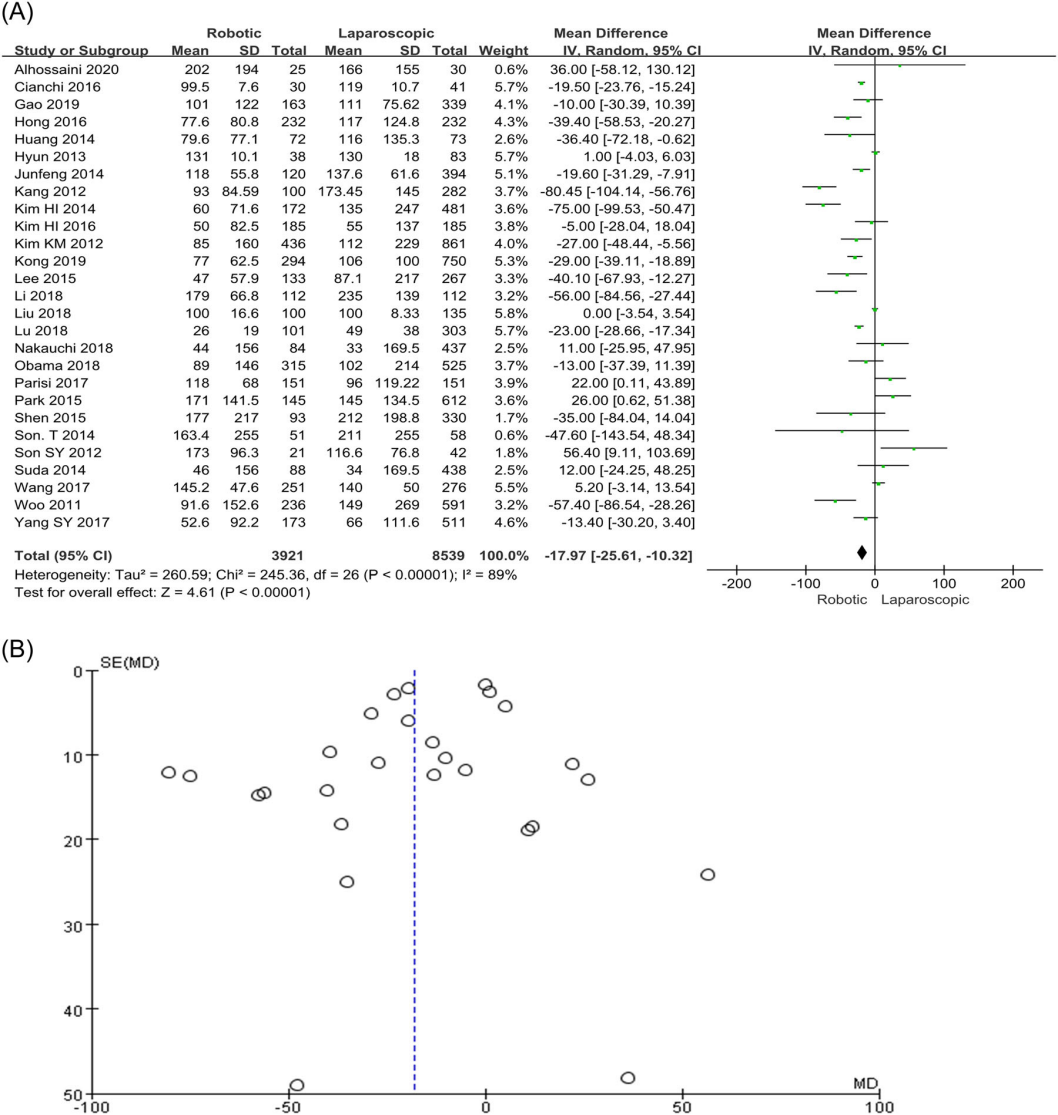
(A, B) Forest graph and funnel graph for blood loss

Meta‐analysis results showed a marked rise in the total amount of blood loss following the LG group compared with RG (MD = −17.97, 95% Cl: −25.61 to 10.32, *p* < 0.001) as shown in Figure 2A,B.

### 3.4 Conversion rate

**Figure 3:**
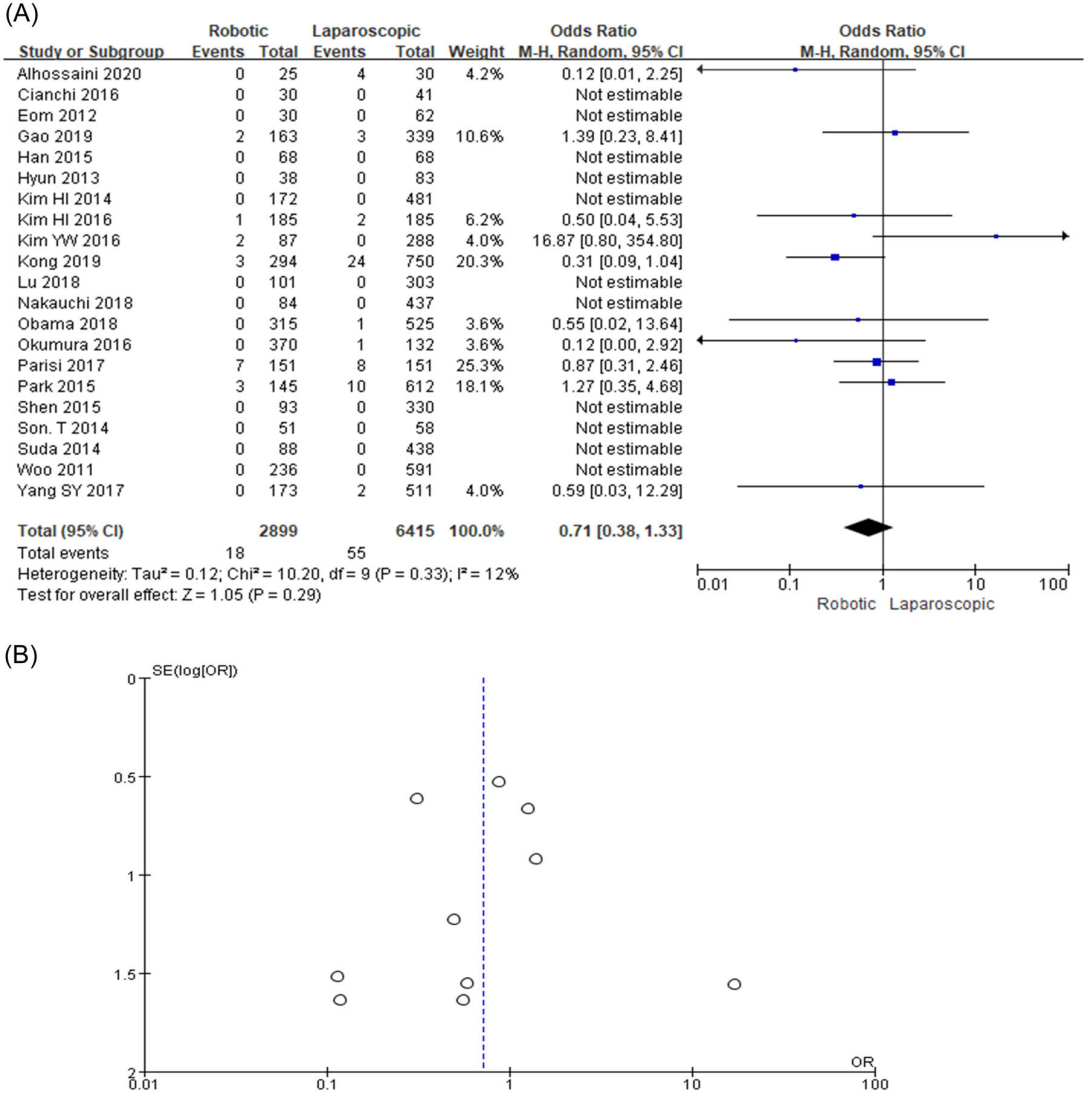
(A, B) Forest graph and funnel graph for conversion rate

The overall conversion rate was 0.6% (18/2899) to open surgery (OS) in the RG group and 0.86% (55/6415) in the LG group. In this study, the conversion rate following OS was statistically not significant in 21 different trials within the two groups (OR = 0.71, 95% CI: 0.38–1.33, *p* = 0.29) as shown in Figure 3A,B.

### 3.5 Overall complication

**Figure 4:**
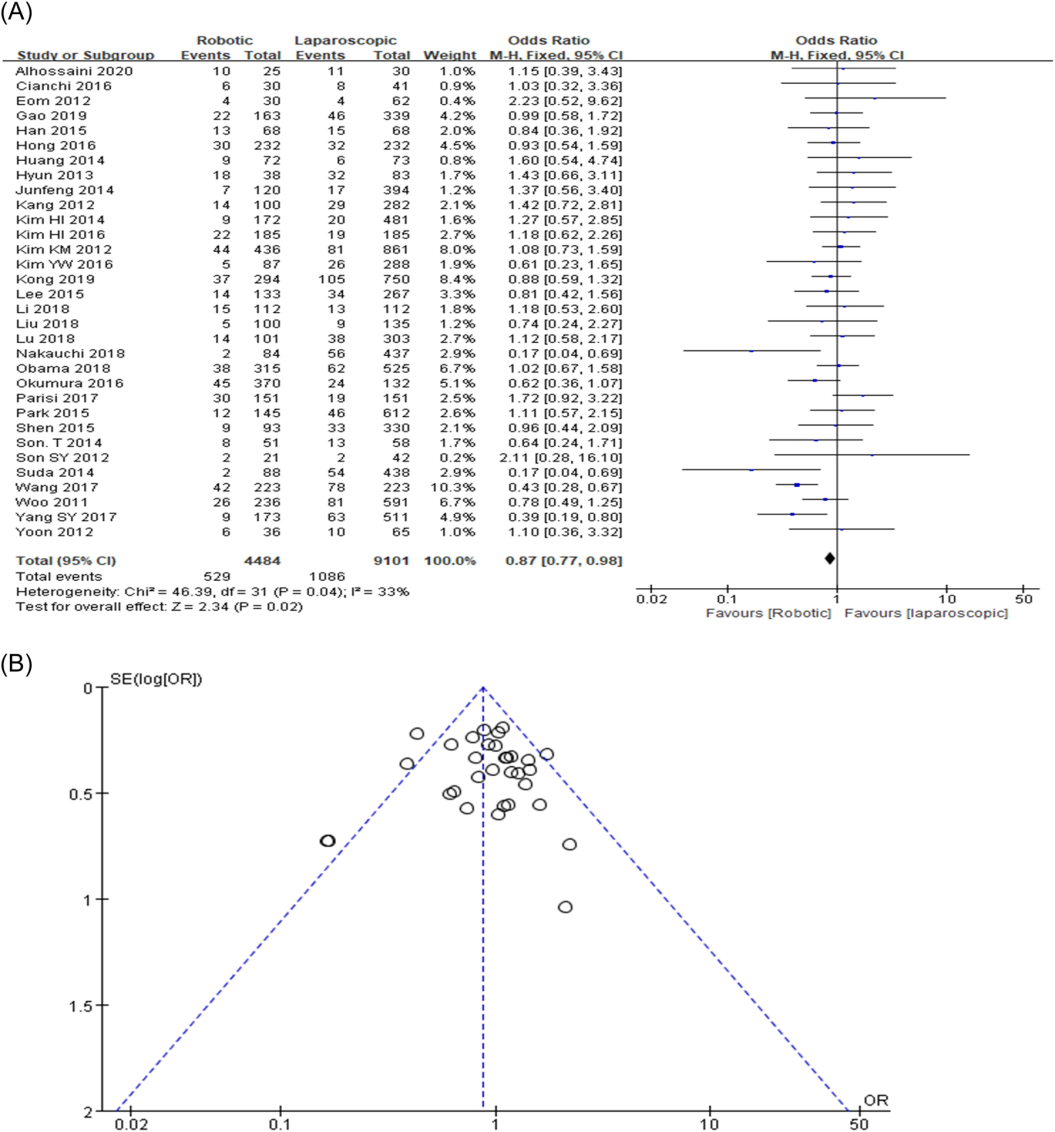
(A, B) Forest graph and funnel graph for overall complication

An overall complication has been found in multiple 32 studies. The proportion rate for overall complications was 11.8% (529/4484) in the RG group and 11.9% (1086/9101) in the LG group. The result for this study proposed a statistically significant (OR = 0.87, 95% CI: 0.77–0.98, *p* = 0.02) as shown in Figure 4A,B.

### 3.6 Clavien–Dindo classification grade Ⅲ

**Figure 5:**
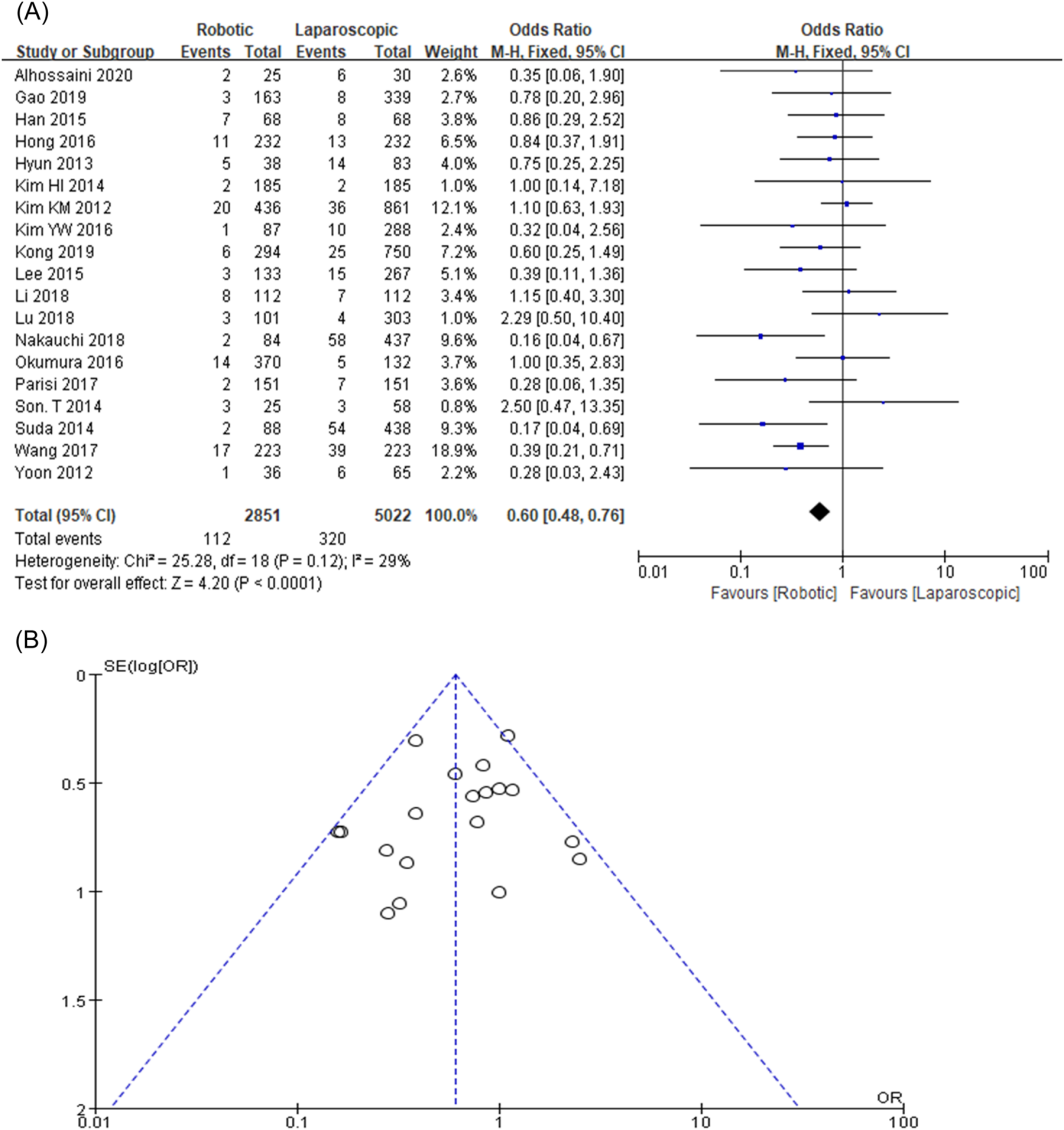
(A, B) Forest graph and funnel graph for Clavien–Dindo grade Ⅲ

The frequency rate of complication in the nineteen retrospective studies reported that Clavien–Dindo grade > Ⅲ in the RG group was 3.9% (112/2851) and LG group was 6.3% (320/5022). The rate is lesser in RG as compared with LG (OR= 0.60, 95% CI: 0.48–0.76, *p* < 0.01) as shown in Figure 5A,B.

### 3.7 Anastomotic leakage

**Figure 6:**
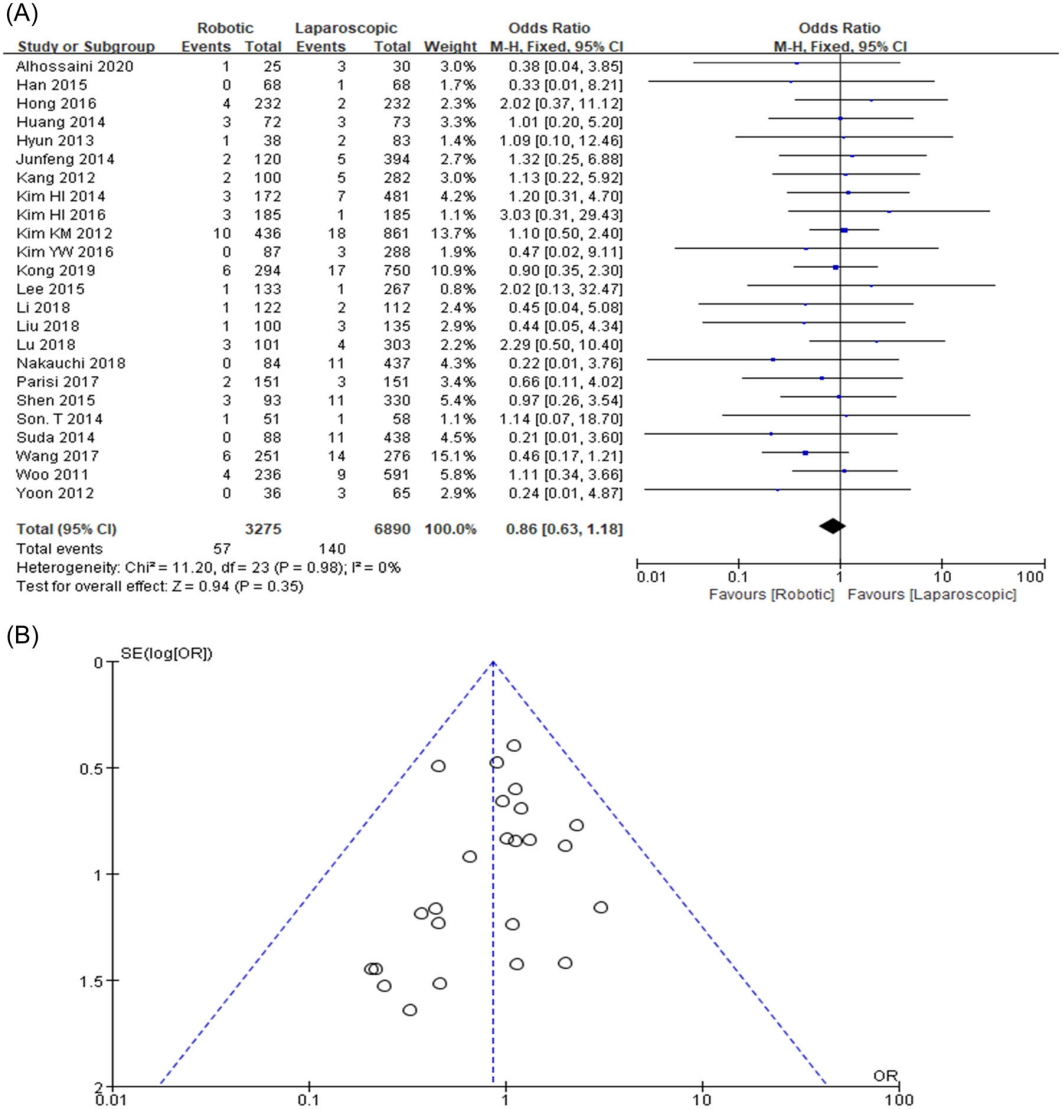
(A, B) Forest graph and funnel graph for anastomotic drip

Overall anastomotic leakage was found in 24 studies. Therefore, the RG group was 1.7% (57/3275) and the LG group was 2.03% (140/6890). Our study did not show the most significant change in the anastomotic leakage (OR = 0.86, 95% CI: 0.63–1.18, *p* = 0.35) as shown in Figure 6A,B.

### 3.8 Distal margin

**Figure 7:**
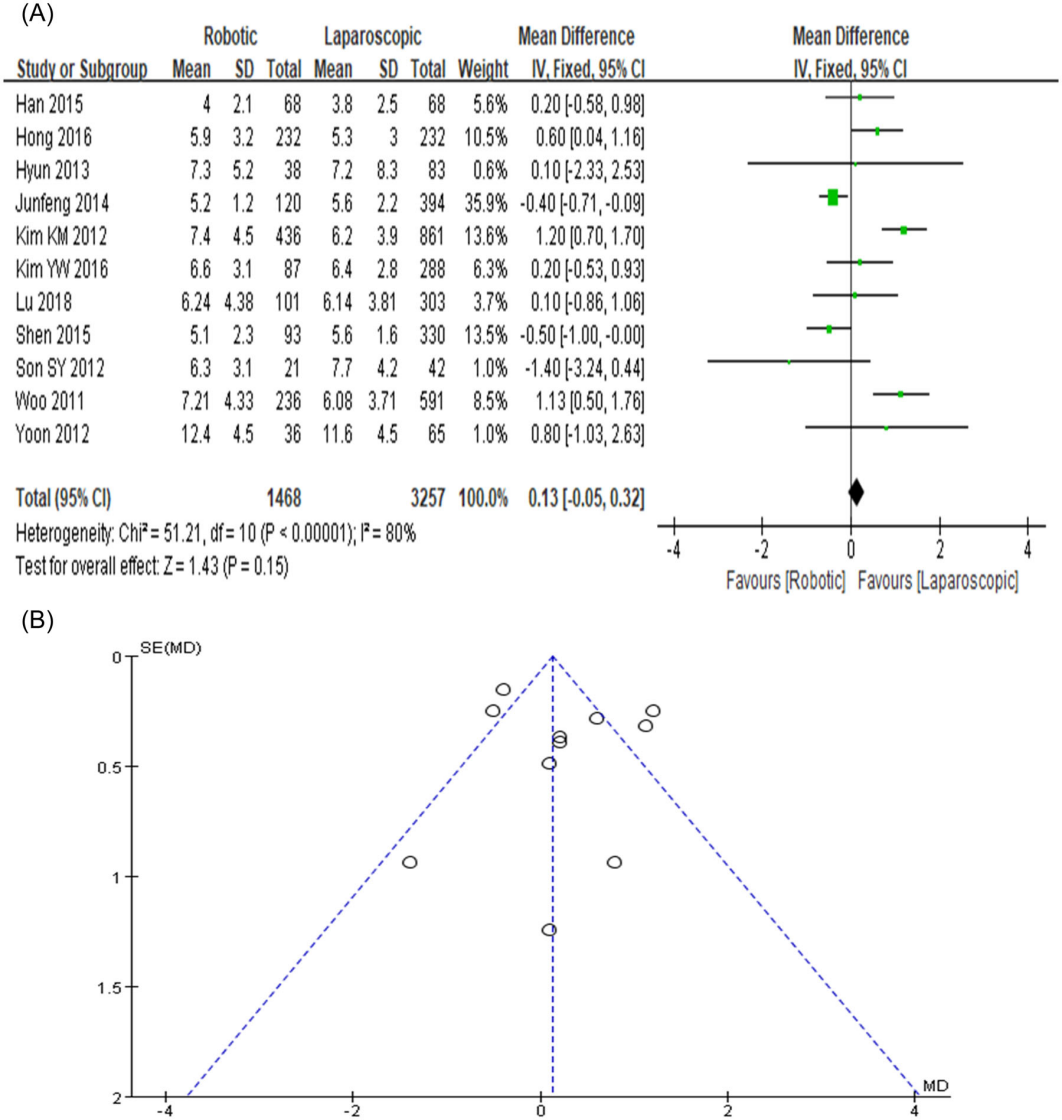
(A, B) Forest graph and funnel graph for distal resection margin

Eleven out of 32 studies informed the DRM. The mean difference in the robotic gastrectomy was found at 6.69 while LG was 6.5. Our study indicated that there is no significant (MD = 0.13, 95% CI: −0.05 to 0.32, *p* = 0.15) as shown in Figure 7A,B.

### 3.9 Proximal margin

**Figure 8:**
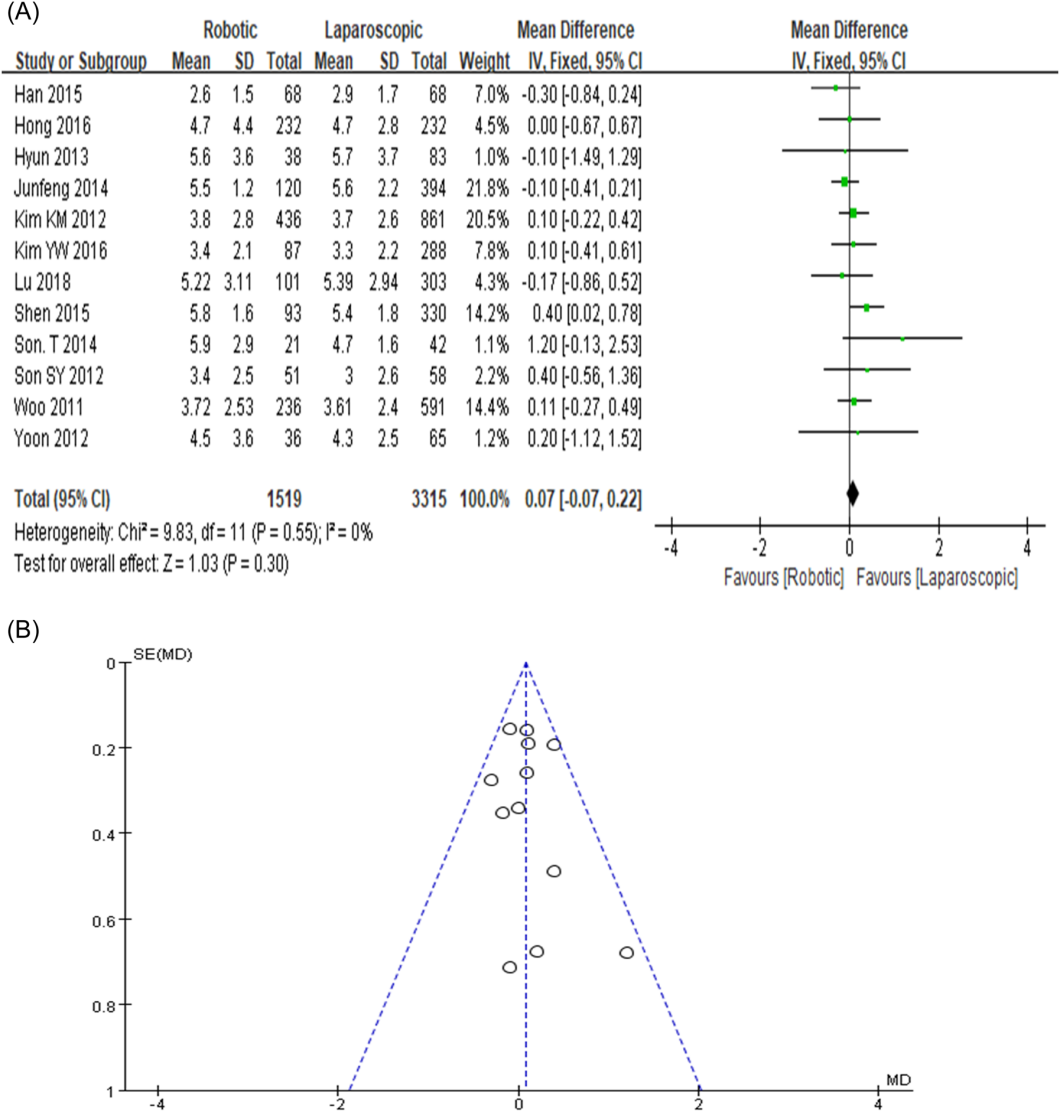
(A, B) Forest graph and funnel graph for proximal resection margin

Following 32 studies the PRM was reported in 12. The mean distance in RG was 4.5 while LG was 4.4. There is no statistical difference seen in RG with comparison to LG group, a mean difference (MD = 0.07, 95% CI: −0.07 to 0.22, *p* = 0.30) as shown in Figure 8A,B.

### 3.10 Harvested lymph node

**Figure 9:**
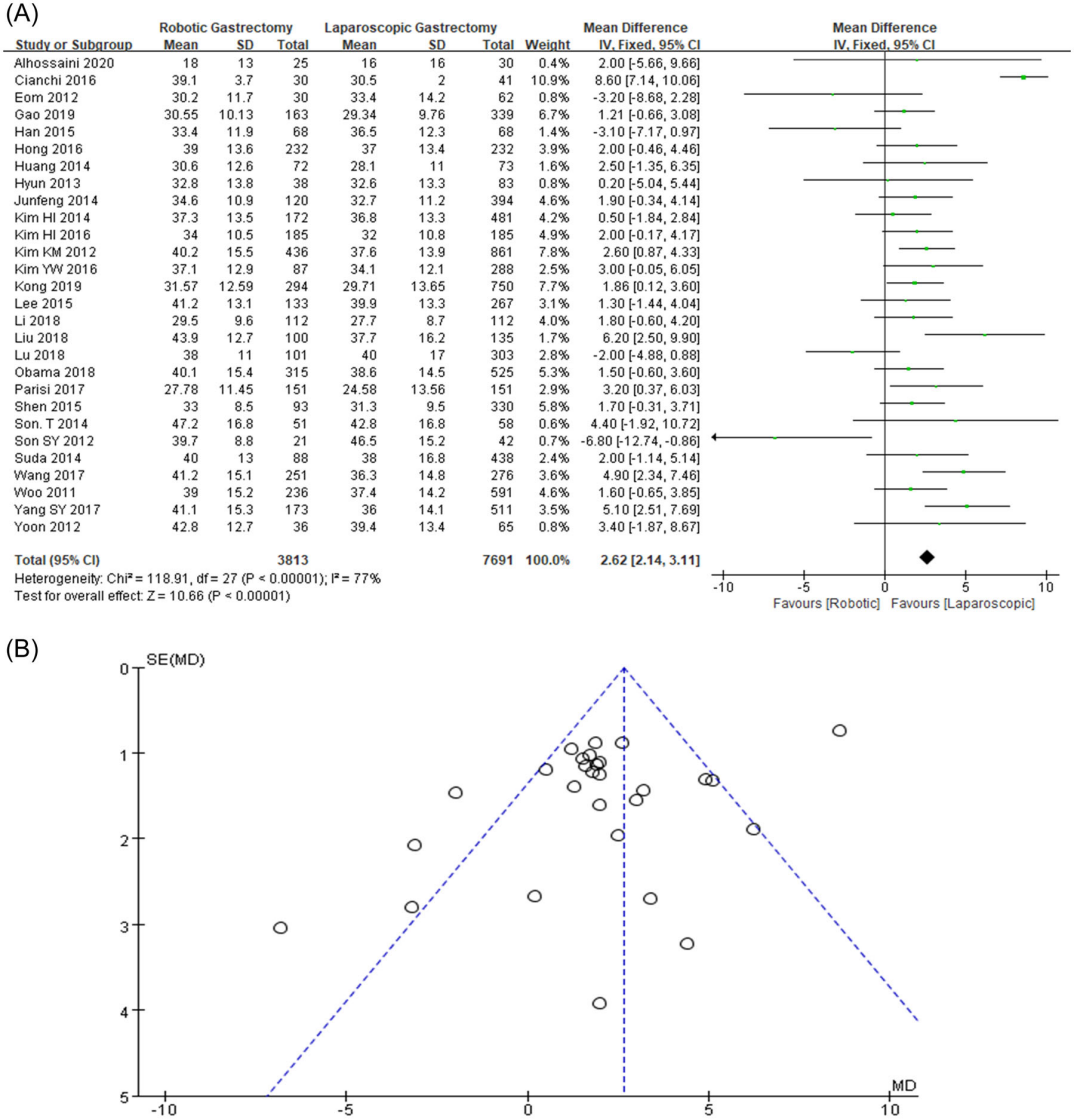
(A, B) Forest graph and funnel graph for the harvested lymph node

Our study reported a raised number of the HLN in RG compared with LG (MD = 2.62, 95% CI: 2.14–3.11, *p* < 0.001). However, our data showed statistically significant as shown in Figure 9A,B.

## 4 DISCUSSION

Over the past years, surgical resection has been the only quality treatment method for gastric cancer. Following the laparoscopic use for gastric carcinoma highly increased in the developing world. Because of certain limitations in laparoscopic surgery, robotic surgery was developed to overcome the practical limitations of laparoscopy. However, robotic surgical resection is still slow due to technical problems, complications, and inefficient procedures.^43^, ^44^, ^45^, ^46^ A recent randomized clinical trial study also described that there is no significant reduction of infectious complications in RG compared with LG for gastric cancer.^47^ Furthermore, fewer studies focus on robotic gastrectomy and LG postoperative complications.^22^, ^48^, ^49^ Therefore, we performed a relevant meta‐analysis and compared the two approaches following the treatment of gastric cancer.

We analyzed the overall complication, blood loss, conversion rate, Clavien–Dindo grade Ⅲ, anastomotic leakage, DRM, PRM, and HLN. Specifically, we find a significant difference in blood loss, Clavien–Dindo grade Ⅲ, and harvested lymph nodes between the two approaches.

Our study informed that the practice of robotic surgery is related to a significant blood loss reduction. Therefore, intraoperative blood loss and the resultant reduced perioperative plasma transfusions are related to improved short‐term clinical management, which shows a correlation to upgraded long‐term oncological consequences.^20^, ^21^, ^22^, ^23^, ^24^, ^33^, ^34^, ^35^, ^36^, ^37^, ^38^, ^39^, ^40^, ^41^, ^42^, ^43^, ^46^, ^47^

Our meta‐analysis exposed that the conversion rate following OS was not significant concerning the necessity for reoperation and postsurgical mortality rate. At the same time, the MIS gastrectomy reported several adhesions, quality precisions to technical difficulties, and extensive damage to adjacent organs.^21^, ^22^, ^23^, ^24^, ^30^, ^31^, ^32^, ^33^, ^34^

Overall complications did not expose any statistically significant outcome. However, the robotic group showed 11.8%, and the laparoscopic group showed 11.9%. We also analyzed the complication according to Clavien–Dindo grade > Ⅲ. It allows us to evaluate the surgical outcomes in medical practice, and this is a simple, objective, reproducible, and good worldwide tool for evaluating postoperative progression. We examined grade Ⅲ postoperative complication as it is the most challenging following the quality of life, clinical assistance, and improved survival. However, our study showed a lower rate in RG of 3.9% compared with LG at 6.3%.^9^, ^11^, ^20^, ^24^, ^39^, ^40^, ^41^, ^42^

This study showed that anastomosis leakage was almost the same in both groups, but our result's statistical value is not significant. In our meta‐analysis, laparoscopic and robotic approaches for DRM were 6.5% and 6.7% and in PRM were 4.4% and 4.5%, respectively. Furthermore, the previous meta‐analysis also described distal and proximal resection margins are not statistically significant but did not provide any specific bias study data.^50^ So, our study concluded that it may be because of the fewer study data as shown in Figures 7B and 8B. Anyhow, still need more clinical studies on it.

The extent of lymph node recovery in the laparoscopic and robotic gastrectomy's statistically significant, but we have seen an increased rate of the harvested lymph node in RGS as compared with LGS.^10^, ^11^, ^19^, ^20^, ^21^, ^22^, ^23^, ^24^, ^25^, ^32^ A previous meta‐analysis also concluded that lymph nodes are more harvested in RG as compared with LG but did not provide specific bias study data on it.^51^ In our meta‐analysis, we concluded that it may be due to a biased study as shown in Figure 9B. as a result, additional clinical trials are required.

In our study, all the articles assessed the comparison between robotic and LG. To our knowledge, this is the first study that specifically compared postoperative outcomes. Though, there are many limitations. All the detailed studies are retrospective and nonrandomized. Variable quantity analysis showed heterogeneity owing to the retrospective analysis's characteristics and the different surgeons used altered surgical skills according to regional dissimilarity. Anyhow, more clinical research on a large scale in postoperative complications is required to know a better outcome for long‐term survival.

## 5 CONCLUSION

It concludes that the practice of robotic gastrectomy is the most feasible and quality technique for gastric carcinoma, with improved surgical outcomes due to harvested lymph nodes, Clavien–Dindo grade Ⅲ, and intraoperative blood loss as compared with LG. However, it still needs to be testified with additional clinical trials. Furthermore, long‐lived oncological consequences must be the main issue for further studies.

## AUTHOR CONTRIBUTION

*Conceptualization, literature review, protocol development, title, and abstract review, full‐text review, data extraction, manuscript writing, revision, and submission*: Muhammad Ali. *Data collection and revision*: Yang Wang and Jianyue Ding. *Study direction and final revision*: Daorong Wang.

## CONFLICT OF INTEREST

The authors declare no conflict of interest.

## TRANSPARENCY STATEMENT

The lead author (manuscript guarantor) affirms that this manuscript is an honest, accurate, and transparent account of the study being reported; that no important aspects of the study have been omitted; and that any discrepancies from the study as planned (and, if relevant, registered) have been explained.

## Data Availability

The data are available via referenced articles. Any further data regarding the article can be made available upon sensible request to the corresponding author.
